# An App for Identifying Children at Risk for Developmental Problems Using Multidimensional Computerized Adaptive Testing: Development and Usability Study

**DOI:** 10.2196/14632

**Published:** 2020-04-16

**Authors:** Chen-Fang Hsu, Tsair-Wei Chien, Julie Chi Chow, Yu-Tsen Yeh, Willy Chou

**Affiliations:** 1 Department of Pediatrics Chi Mei Medical Center Chi Mei Medical Groups Tainan Taiwan; 2 Department of Medical Research Chi Mei Medical Center Chi Mei Medical Groups Tainan Taiwan; 3 Department of Pediatrics Taipei Medical University Chi Mei Medical Groups Taipei Taiwan; 4 Medical School St George’s, University of London London United Kingdom; 5 Department of Physical Medicine and Rehabilitation Chi Mei Medical Center Chi Mei Medical Groups Tainan Taiwan; 6 Department of Physical Medicine and Rehabilitation Chung Shan Medical University Taichung Taiwan

**Keywords:** computer adaptive testing, developmental delay, multidimensional, mobile phone, screening

## Abstract

**Background:**

The use of multidomain developmental screening tools is a viable strategy for pediatric professionals to identify children at risk for developmental problems. However, a specialized multidimensional computer adaptive testing (MCAT) tool has not been developed to date.

**Objective:**

We developed an app using MCAT, combined with Multidimensional Screening in Child Development (MuSiC) for toddlers, to help patients and their family members or clinicians identify developmental problems at an earlier stage.

**Methods:**

We retrieved 75 item parameters from the MuSiC literature item bank for 1- to 3-year-old children, and simulated 1000 person measures from a normal standard distribution to compare the efficiency and precision of MCAT and nonadaptive testing (NAT) in five domains (ie, cognitive skills, language skills, gross motor skills, fine motor skills, and socioadaptive skills). The number of items saved and the cutoff points for the tool were determined and compared. We then developed an app for a Web-based assessment.

**Results:**

MCAT yielded significantly more precise measurements and was significantly more efficient than NAT, with 46.67% (=(75-40)/75) saving in item length when measurement differences less than 5% were allowed. Person-measure correlation coefficients were highly consistent among the five domains. Significantly fewer items were answered on MCAT than on NAT without compromising the precision of MCAT.

**Conclusions:**

Developing an app as a tool for parents that can be implemented with their own computers, tablets, or mobile phones for the online screening and prediction of developmental delays in toddlers is useful and not difficult.

## Introduction

Preschooler developmental delay has been defined to occur when a child does not reach developmental milestones, including gross motor, fine motor, language, cognitive, and social skills, at the expected times [1] or when a child’s developmental milestones appear more slowly compared to those of typically developing children [2]. There is usually a more specific condition causing this delay, such as fragile X syndrome or other chromosomal abnormalities. However, it is sometimes difficult to identify the underlying condition [3].

Substantial variations in the prevalence of developmental delay have been reported, including 5.7%-7.0% in Norwegian infants [4], 3.3% in American children [5], and 6%-8% in Taiwanese preschoolers [6]. Some methodologies do not facilitate comparison of prevalence rates because of differences in case definitions and criteria, type of measures used, age, and whether the studies included low- or high-risk populations [4]. Therefore, more standardized developmental screening tools are required [7].

### Increase in Screening Rate

In 2001, the American Academy of Pediatrics (AAP) recommended that all children undergo standardized developmental screening as part of their well-child care [8]. However, there are barriers preventing pediatricians from using such screening tools, including lack of personnel, time, or effective screening tools [9]. Therefore, busy practitioners (or parents) should be provided with a quick, simple, valid, and reliable screening tool to allow for quick and efficient screening [10].

Between 1994 and 2002, only 23%-30% of pediatricians screened their patients for developmental delays [11,12]. After a series of enhanced research and educational programs were launched and such screening tools were recommended, there has been an upward trend in the use of screening, reaching up to 48% in 2009 [9] and exceeding 90% in 2011 [13,14] in the United States.

### Need for Efficiency and Precision

Many types of screening tools have been designed to detect possible global developmental problems [15-20] and to provide a quick overview of the development of children’s communication, gross and fine motor, social, and problem-solving skills. Choosing an appropriate and age-matched checklist for parents to fill out is an added burden.

A search of PubMed on November 13, 2019 with the term “multidimensional computerized adaptive testing” (MCAT) yielded 45 articles, and searching with the term “computerized adaptive testing” (CAT) yielded 483 articles. By the end of 2019, more than 8674 abstracts were retrieved from the PubMed database using the search term “cutoff point.” However, none of these articles discussed methods of determining the cutoff points for CAT (or MCAT) in the use of screening tools for assessing developmental delay in children.

### Using a Multidimensional Developmental Screening Tool

Although the Multidimensional Screening in Child Development (MuSiC) tool for children 0-3 years old has been reported [7], to our knowledge, there is no available online app for screening that is used in clinical practice. Therefore, a multidomain developmental screening tool is urgently needed [21,22].

In this study, we investigated the feasibility of screening toddlers (1- to 3-year olds) using the MCAT combined with MuSiC for toddlers, including (i) comparisons with MCAT and nonadaptive testing (NAT; responding to all items) in efficiency and precision using a Monte Carlo simulation method, (ii) determining cutoff points for a variety of ages and stages using a parent-completed child monitoring system, and (iii) developing an online MCAT app for mobile phones to efficiently collect data and discriminate developmental delays for preschoolers.

## Methods

### Study Data: Item Difficulty and Person Measures

After retrieving 75 item parameters from the MuSiC literature item bank [7] for 1- to 3-year-old children, we simulated 1000 person measures from a normal standard distribution to compare the efficiency and precision of MCAT and NAT in five domains: cognitive skills, language skills, gross motor skills, fine motor skills, and social skills (see Multimedia Appendix 1).

Based on the maximum reported range of the released item difficulties from –7.35 to 8.03 [7], person measure true scores were set in the range of –8 to 8 logits (log odds). Applying the study’s cutoff points (mean –7.366, cognitive skills –4.85, language skills –7.44, gross motor skills –9.95, ﬁne motor skills –6.15, and social skills –8.44) in logits for the 137 participants (2-year-old children) [7], the highest skill level was found to be in the cognitive domain and the lowest was in the gross motor domain. The lower the score, the greater the developmental delay. Finally, we used Rasch [23] ConQuest software for calibrating item difficulties for these five domains in the tools.

As the reliability of a scale (ie, Cronbach alpha) increases, so does the person-number of ranges that can be confidently distinguished [24-27]. Measures with a reliability of 0.67 will vary within two groups, those of 0.80 will vary within three groups, and those of 0.90 will vary within four groups [24].

### Simulating Person Response to Items Across Domains

**Figure 1:**
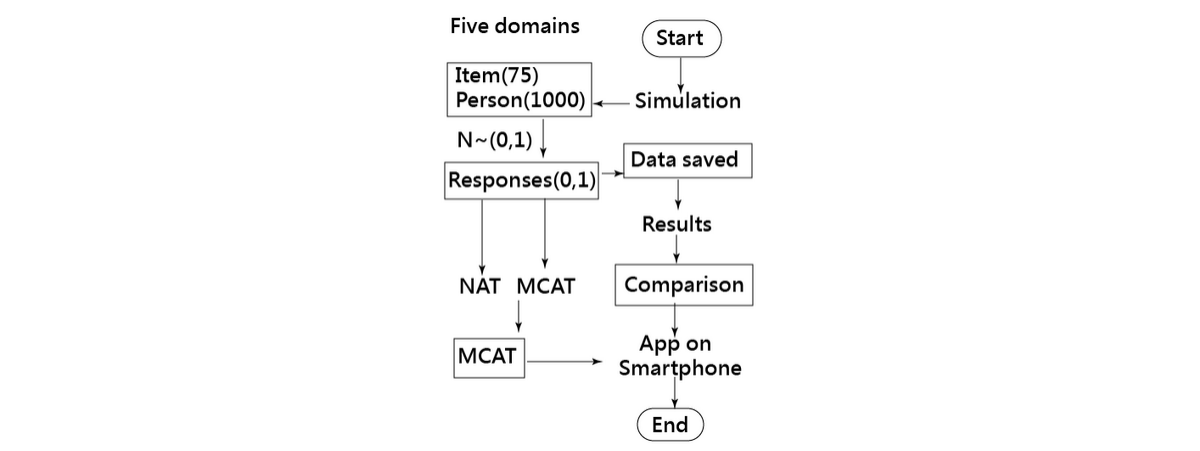
Study flowchart.

When the person abilities and item difficulties are known, as described above, the responses can be obtained in a rectangle 1000 × 75 matrix form that contains the five domains using a Rasch simulation computer process [28]. Therefore, the first study aim of comparing the efficiency and precision of MCAT and NAT can be assessed using a Monte Carlo simulation method (Figure 1 and Multimedia Appendix 2).

### Design of the App

#### Algorithm Using Rasch Analysis for Considering Item Difficulties

In classical test theory, the summation score (or the linear transformed score such as a T score) is often used as the latent trait estimation (ability=success rate) under the condition that all item difficulties are equal (ie, have a common weight). The item response theory (IRT)-based Rasch model [23] was developed to deal with the real-world scenario that not all item difficulties are equal.

All person measures and item difficulties were compared using a common scale unit in logit. The person (n) probability of answering a specific item (i) is denoted by the formula: Prob_ni_=exp (ability_n_–difficulty_i_)/(1+exp [ability_n_–difficulty_i_]). If all item difficulties are known, all possible likelihood values can be obtained using the formula II*p_ni_* (ie, multiplying all probabilities across items) and using a range of possible abilities from –8 to 8 logits. This is the principle of CAT using the two known conditions (ie, item difficulties and person responses to items) to estimate the person measure. All person measures and item difficulties are on an interval continuum [29]. Two other requirements are that items should be unidimensional and locally independent when CAT is applied; otherwise, the estimation will not be precise.

#### Cutoff Points Used for Multidimensional Screening in Child Development

To determine the overall global level of developmental delay, we first computed the number of the strata based on subscale reliability, and then referred to the Rasch threshold difficulty guideline [30] to optimize an appropriate distance between two thresholds in the range of 1.4-5.0 logits for all separated groups with an equal sample size.

As suggested by Maslach et al [31,32], an equal sample size in each stratum was applied to determine the cutoff points. Accordingly, a threshold at zero logits is suggested for two strata; –0.7 and 0.7 {1.4 logit difference with probabilities at 0.33 and 0.67=1–exp(–0.7)/(1+exp[–0.7])} for three strata; –1.1, 0.0, and 1.1 {1.1 logit difference with probabilities at 0.25, 0.50, and 0.75=1–exp (–1.1)/(1+exp[–1.1])} for four strata; and –1.4, –0.4, 0.4, and 1.4 {1.0 logit difference with probabilities at 0.20, 0.40, 0.60, and 0.80=1–(–1.4)/(1+exp[–1.4])} for five strata. Therefore, the second study aim of determining cutoff points is possible.

#### Multidimensional Computer Adaptive Testing Used on a Developmental Screening Tool

The multidimensional random coefficients multinomial logit model (MRCMLM) has been proposed to capture the complexity of modern assessments [33,34]. The merging of MRCMLM and CAT, or other multidimensional IRT models and CAT, is called multidimensional computerized adaptive testing (MCAT) [35]. We can consider using MCAT to simultaneously estimate person measures for an inventory consisting of multiple subscales such as the developmental screening tool developed in this study [7]. We programmed an online MCAT using maximum-likelihood estimation with the Newton-Raphson iteration method to administer the 5-domain developmental screening tool.

We applied MCAT stop rules as described previously [36], such as when the person reliability for each domain reaches a specific level; for example, 0.80=[1SEM_pi_^2^]=10.44^2^], where SEM_pi_=person standard error of measurement on item i=1/variance_pi_=1/information_pi_, and the last three average consecutive person estimation changes are <0.05 in residual difference between the two stages in the CAT process after the minimal necessarily completed number of items on each domain is 3. The final graphical representation is shown with items in domain order on a mobile phone. Therefore, the third study aim for online MCAT development is also possible (see the video in Multimedia Appendix 2).

### Data Analysis and Website Design

ConQuest Rasch software [37] was used to calculate parameters on the five subscales of response datasets. The variance-covariance and correlation matrices in relation to the ﬁve domains were extracted from tables in ConQuest (see Multimedia Appendix 3). Independent *t* tests were used to compare the efficiency and precision of NAT and MCAT. Significance was set at *P*<.05 (two-tailed).

### Availability of Data and Materials

This research is based on a simulation study. All codes and data can be obtained from the Multimedia Appendix files of this study.

## Results

### Analyses of Domains and Items

**Figure 2:**
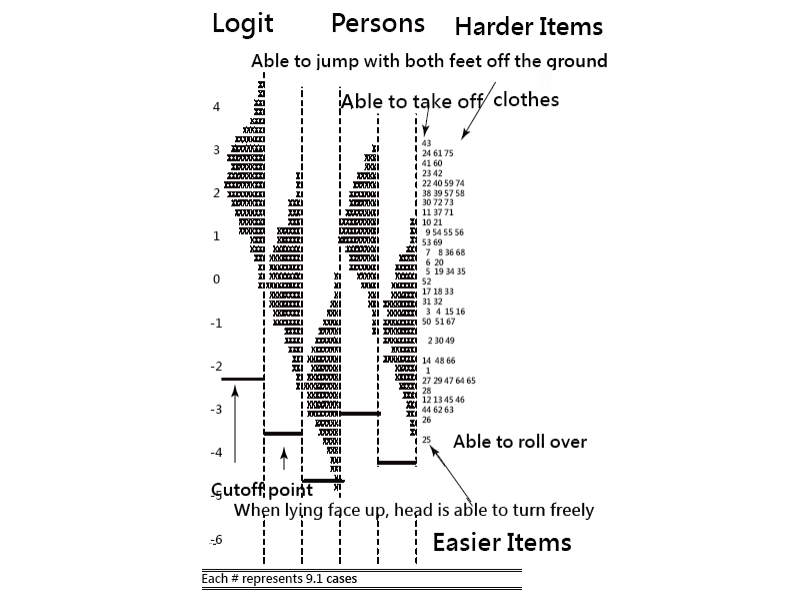
Multidimensional analysis of dispersions of persons (first 5 columns) and items (last column) across domains.

Figure 2 shows the dispersed person measures and item difficulties, demonstrating that the different means of the five domains are significantly located upward and downward on the left side of the dispersion. Correlation coefficients were highly consistent among the five domains in person measures (Table 1). All person reliabilities showed a correlation coefficient >.8, indicating three person strata separated in this sample [24].

**Table 1:** Variance-covariance matrix (plus correlation matrix and reliability) for the ﬁve domains.^a^

Category		Cognitive	Language	Gross motor	Fine motor	Social
**Domain skill**						
	Cognitive		0.95	0.95	0.85	0.98
	Language	0.93		1.05	0.96	1.07
	Gross motor	0.93	0.94		0.96	1.09
	Fine motor	0.91	0.93	0.94		0.99
	Social	0.92	0.92	0.94	0.93	
Variance		0.94	0.12	1.11	0.94	1.21
Reliability		0.84	0.85	0.86	0.86	0.85

^a^The bottom left diagonal shows correlation coefficients; the right top diagonal shows covariance.

### Comparison of Efficiency and Precision Between Nonadaptive Testing and Multidimensional Computer Adaptive Testing

Significantly (*P*<.001) fewer items were answered on MCAT than on NAT without compromising its precision (*P*=.22). The efficiency of MCAT was a 46.67% (=(75-40)/75) savings in item length. The average means of items used across domains in MCAT were 6, 6, 10, 10, and 8 for cognitive, language, gross motor, fine motor, and social domains, respectively. There were significant differences in item length across domains between NAT and MCAT (Table 2).

**Table 2:** Comparisons of item length and skill ability on domains between nonadaptive testing (NAT) and computerized adaptive testing (CAT).

Category		Cognitive		Language		Gross motor		Fine motor		Social		*P* value	
**Item length**													
	NAT		11		13		19		18		14		
	CAT		6		6		10		10		8		.01
**Skill ability**													
	NAT		0.088		0.15		0.065		0.021		0.032		
	CAT		0.086		0.067		0.023		0.023		0.033		.07

### Cutoff Points Used for Multidimensional Screening in Child Development

The person strata could be separated into three subgroups. The global cutoff points were determined at –0.7 and 0.7 logits using the criterion of averaging all domain logit scores. Each stratum had an equal accumulated probability of 0.33. The original domain cutoff points for 24-month-old children are shown in Figure 2.

### Online Multidimensional Computer Adaptive Testing Assessment

**Figure 3:**
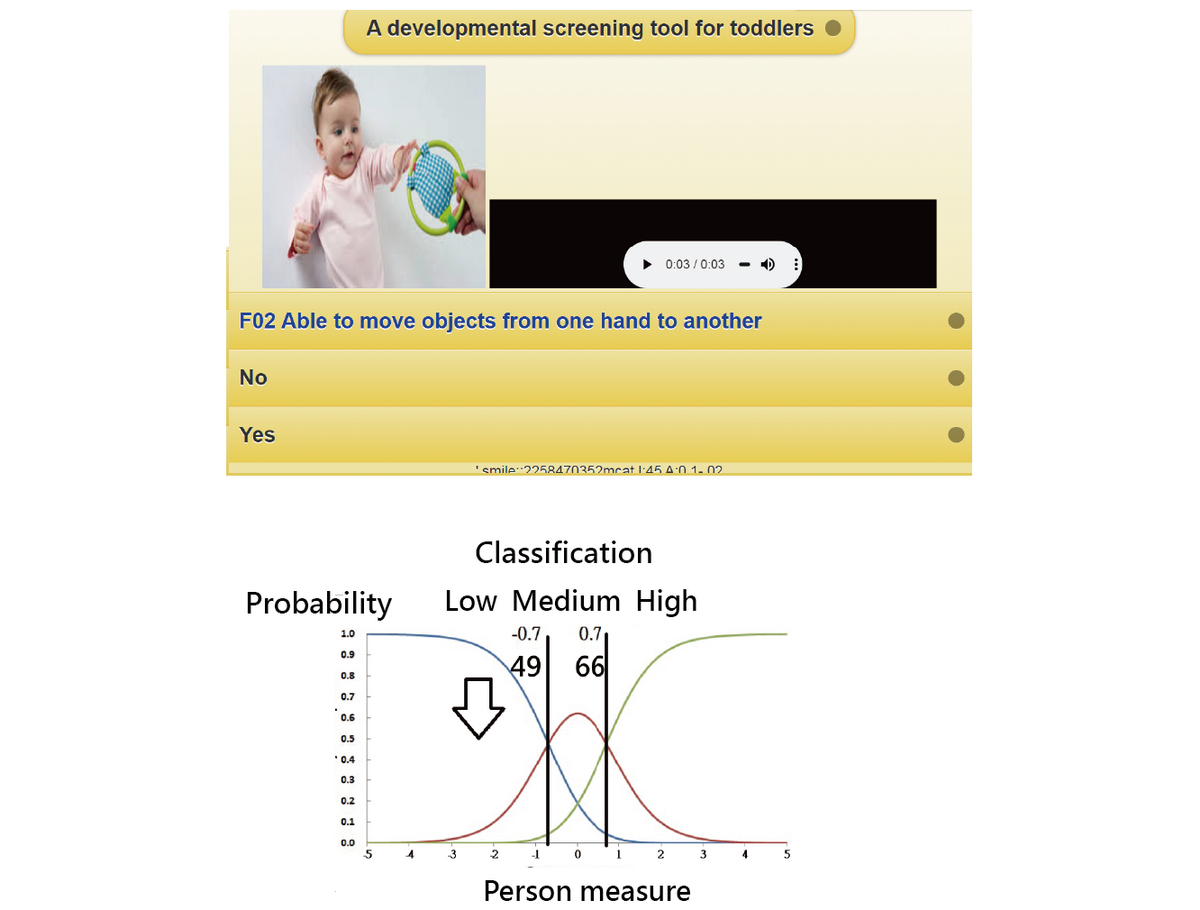
The online process of MCAT on a mobile phone.

Scanning a Quick Response (QR) code (Figure 3) or downloading the app will cause the MuSiC developmental delay questionnaire to appear on the mobile phone. We developed an MCAT mobile survey procedure to demonstrate our newly designed MuSiC application in action. The assessment used audio and video to process each child item-by-item (Figure 3, top left). Person domain scores can be estimated using MCAT (Figure 3).

In the MCAT process, adaptive item selection is based on maximizing the determinant of the provisional information matrix across unanswered items. The measurement of standard error for each subscale decreased when the number of items increased (Figure 3). The result with person measures across all domains instantly displays on the mobile phone (Figure 3). The global cutoff points shown in Figure 3 can serve as a guide to roughly check the level of developmental delay for the child at a low, medium, or high location. The detailed cutoff point for a specific age can be determined using Figure 2 to assess whether a follow-up stage that requires a re-examination of development delay is reached or to refer to the indicator for which any specific item should be passed but failed for the age.

## Discussion

### Principal Findings

We verified that (1) the number of answered items is significantly lower (*P=*.01) on MCAT than on NAT without compromising its precision (*P=*.07), (2) the global cutoff points should be set to –0.7 and 0.7 logits to separate persons into equal size groups (*P*=.33 each) (cutoff points for 24-month-olds are shown in Figure 2), and (3) an available-for-download online MCAT app for parents is suitable for mobile phones.

### Contribution to Existing Research

We verified that CAT [38,39] (or MCAT [34-36]) is more efficient than NAT, which is consistent with the literature. We also confirmed that, without compromising its measurement precision, MCAT-based MuSiC requires significantly fewer questions to measure developmental delay for children compared with NAT. MCAT is more efficient than NAT, especially in cases of high correlation among measures and more dimensions [33-35]. However, this is the first online MCAT app reported to date.

Twenty-one pieces of Ages & Stages Questionnaires (ASQ-3)—a parent-completed child monitoring system) [20]—were developed to be used for children aged 2, 4, 6, 8, 9, 10, 12, 14, 16, 18, 20, 22, 24, 27, 30, 33, 36, 42, 48, 54, and 60 months old. Thus, we should develop 21 item pools (eg, 21 tests) and domains for each age by mimicking the use of MCAT in this study to screen for developmental delays. If the child’s age is known at the start of the screening, MCAT can estimate the person measure and show the cutoff points in a diagram (Figure 3) along with a judgment (pass or fail) according to specified items for the age as previously described for methods used in Taiwan [15-17].

If at least one developmental delay is found in one of the domains, the child should be sent to a hospital for a medical examination because MCAT covers multiple domains with tailored items for an individual child, which is expected to increase assessment precision. MCAT considers item difficulties and correlations between domains. In contrast, the ASQ-3 contains only six items in each domain, which reduces the instrument’s reliability because of the short items and ignored item weights. This sacrifices assessment precision because of a large amount of measurement error.

### Implications for Change

In 2001, the AAP recommended that all children undergo standardized developmental screening as part of their well-child care [8] and hoped for all children to have access to a standardized, quick, simple, valid, and reliable developmental screening tool [8], along with the rapid development of computer technologies, such as an app for identifying children at risk for developmental problems.

There has been no discussion on methods for determining the cutoff points for CAT (or MCAT) because not all items are endorsed, making it impossible to obtain summation scores in practice. Here, two types of MCAT cutoff points are demonstrated: (1) global cutoff points (set at –0.7 and 0.7) to separate the sample into three equally sized groups (Figure 3), and (2) item-by-item cutoff points (Figure 2) that show whether there is any developmental delay by identifying specific items that the child failed to pass for their age.

### Strengths of This Study

In the MCAT, we included several useful indicators that work well with a Rasch model and CAT. First, the greater the number of difficult items correctly answered by a person, the higher their performance level will be, because the adjustment depends on the residual of the response (ie, observed score – expectation) using the Newton-Raphson iteration method. Second, the outfit mean square error ([Σ^2^ -score]/L=(Σ [residual/standard deviation]^2^)/L, where L=item length) is a macroaberrant behavior indicator that detects whether a person responds with a reasonable behavior pattern to the items [34]. Third, a z-score (residual/standard deviation) is used as a microaberrant response indicator that detects whether the item response is in an acceptable range (ie, |Z|>2.0 [30]) in line with the person’s provisional skill level. All of these indicators, which benefit the interpretation of responses, are rarely seen in classical test theory.

We used ConQuest to estimate the parameters, which is reported to accurately estimate both item and person parameters in multidimensional Rasch models [32,34,37]. The process can be recommended for future studies on the parameter estimation of MCAT.

### Limitations and Future Studies

This study has some limitations. First, the study data were retrieved from published papers [7]. If any parameter was incorrectly embedded, the MCAT would be problematic in practice. Therefore, the MCAT module should be reexamined by many future studies. Second, we determined any cutoff points for age groups in this study. The cutoff point criteria were determined on a theoretically logical basis of an interval latent trait continuum in a logit unit. That is, all abilities within a domain were incrementally increased by the number of logits appropriate for each particular age increase. Future studies are recommended for cutoff point determination across ages in domains for the ASQ-3 or to refer to the indicator for any specific item that should be passed but failed for the age. Third, Figure 2 indicates that some gaps should be filled with missing items, and that more difficult and easier items should be added to the top and bottom areas. The MCAT items were merely extracted from three screening tools commonly used in Taiwan [15-17]. To improve the MuSiC item bank, more appropriate items used in other developmental delay screening tools such as the ASQ-3 should be considered [18]. Fourth, Yes/No items were used in the study. For a more accurate estimate, Yes/Sometimes/Not Yet items, which are used in the ASQ-3, should be investigated in future studies. Finally, the MuSiC item pool was originally used for 1- to 3-year-old children. Future studies are recommended to expand the item pool to include a wider age range in practice.

### Conclusions

Although the MCAT had significantly fewer items than the NAT, the precision of MCAT was not compromised. The online MCAT with a mobile phone facilitates screening for developmental delays in toddlers.

## Appendix

Multimedia Appendix 1 Data in MS Excel format.

Multimedia Appendix 2 Link to online assessment for the MCAT video.

Multimedia Appendix 3 Link to ConQuest.

## Abbreviations

AAP: American Academy of Pediatrics
ASQ-3: Ages & Stages Questionnaires
CAT: computer adaptive testing
IRT: item response theory
MCAT: multidimensional computer adaptive testing
MuSiC: Multidimensional Screening in Child Development
MRCMLM: multidimensional random coefficient multinomial logit model
NAT: nonadaptive testing
QR: Quick Response
