# Supplementary material for: An App for Identifying Children at Risk for Developmental Problems Using Multidimensional Computerized Adaptive Testing: Development and Usability Study
Source: JMIR Pediatr Parent. 2020 Apr 16;3(1):e14632. doi: 10.2196/14632 (PMC7193438; doi:10.2196/14632)
Supplement: Multimedia Appendix 2 [file pediatrics_v3i1e14632_app2.docx]

**Multimedia file 2:**

Online assessment for the MCAT video at http://www.healthup.org.tw/marketing/course/information/MCAT_toodler2.mp4
