# Supplementary material for: An App for Identifying Children at Risk for Developmental Problems Using Multidimensional Computerized Adaptive Testing: Development and Usability Study
Source: JMIR Pediatr Parent. 2020 Apr 16;3(1):e14632. doi: 10.2196/14632 (PMC7193438; doi:10.2196/14632)
Supplement: Multimedia Appendix 3 [file pediatrics_v3i1e14632_app3.docx]

**Multimedia file 3:**

ConQuest was used in this study

http://www.healthup.org.tw/marketing/course/information/MCAT_toodler_conquest.mp4
